# Behavioural change models for infectious disease transmission: a systematic review (2010–2015)

**DOI:** 10.1098/rsif.2016.0820

**Published:** 2016-12

**Authors:** Frederik Verelst, Lander Willem, Philippe Beutels

**Affiliations:** 1 Centre for Health Economics Research and Modelling Infectious Diseases, Vaccine and Infectious Disease Institute, University of Antwerp, Antwerp, Belgium; 2 School of Public Health and Community Medicine, The University of New South Wales, Sydney, New South Wales, Australia

**Keywords:** behaviour, model, infectious disease, vaccination, game theory, individual-based

## Abstract

We review behavioural change models (BCMs) for infectious disease transmission in humans. Following the Cochrane collaboration guidelines and the PRISMA statement, our systematic search and selection yielded 178 papers covering the period 2010–2015. We observe an increasing trend in published BCMs, frequently coupled to (re)emergence events, and propose a categorization by distinguishing how information translates into preventive actions. Behaviour is usually captured by introducing information as a dynamic parameter (76/178) or by introducing an economic objective function, either with (26/178) or without (37/178) imitation. Approaches using information thresholds (29/178) and exogenous behaviour formation (16/178) are also popular. We further classify according to disease, prevention measure, transmission model (with 81/178 population, 6/178 metapopulation and 91/178 individual-level models) and the way prevention impacts transmission. We highlight the minority (15%) of studies that use any real-life data for parametrization or validation and note that BCMs increasingly use social media data and generally incorporate multiple sources of information (16/178), multiple types of information (17/178) or both (9/178). We conclude that individual-level models are increasingly used and useful to model behaviour changes. Despite recent advancements, we remain concerned that most models are purely theoretical and lack representative data and a validation process.

## 1. Introduction

Infectious diseases can have a large impact on society as they can negatively affect, among others, morbidity, mortality, unemployment and inequality. As a result, prevention and control of infectious diseases are important for public health and welfare.

The main objective of infectious disease transmission models is to inform and guide policy-makers to prepare for and respond to (re)emerging infectious diseases, particularly when sufficient information from controlled experiments is lacking. However, the impact of infectious disease transmission and policy interventions are subject to hosts' behaviour. Therefore, there is an interest to incorporate behaviour change in response to disease-related information into models for infectious disease transmission.

Numerous historical infectious disease experiences confirm the existence of a so-called behavioural immune system [1] in humans. For example, during the 2003, severe acute respiratory syndrome (SARS) outbreak people took precautionary actions such as wearing face masks, hand-washing, avoiding public transport, restaurants, shops and other crowded places in Hong Kong [2,3] and Beijing [4]. In addition, the 2009 A/H1N1 influenza pandemic has triggered a significant proportion of the population to adapt their behaviour and take preventive measures such as social distancing [5,6].

We refer to models incorporating behavioural immunity as ‘behavioural change models’ (BCMs), which typically complement models for disease transmission in an attempt to mimic real life dynamics. In essence, a BCM is a model in which individuals are responsive to external information about the disease and as a result take one or more preventive measures to reduce the chance of contracting the disease. The external information individuals respond to can be global (equally available and relevant to all individuals) or local (individual availability and relevance determined by physical or social proximity to the information source). Furthermore, this information can be specified in terms of actual risks (‘prevalence-based’) or of perceptions of these risks (‘belief-based’), as well as a mixture of all the above [7]. Vaccination is a common prevention measure with varying uptake, given historical fluctuations in the trade-off between the perceived risks of vaccine-related side effects (VRSEs) and of vaccine-preventable disease. Other common prevention measures include social distancing and condom use.

A widely used theoretical foundation for the formation and dynamic nature of individuals' behaviour comes from game theory. Game theory has a rich history in social sciences with the Prisoner's Dilemma being a frequently used illustration (see [8] for a comprehensive introduction). Game theory assumes individuals take rational decisions based on a trade-off that embodies the anticipated rational decisions of all other individuals in society. Even though these assumptions are often not observed in real life [9], a multitude of BCMs in the setting of infectious disease transmission still use a game-theoretical foundation that caused the development of, for instance, ‘vaccination games’ [10] and ‘epidemic games with social distancing’ [11].

Another foundation for behaviour change is found in the fields of network science and individual-based modelling (IBM), where there are opportunities to develop more realistic models by introducing (more) heterogeneity. The challenge here is to find a balance between model complexity and computational boundaries. Some examples of behavioural change research for which network science has been used include models using adaptive contact networks [12], vaccinating behaviour in social contact networks [13] and social distancing in sexual contact networks [14].

Although there is increased recognition for the need to incorporate behavioural changes in infectious disease transmission models, a consensus on the proper methodology to do so is lacking. It appears much research is not supported by empirical information but departs from a theoretical foundation with arbitrarily chosen parameter values and no validation process. As a result, there is large heterogeneity in the triggers for behavioural change and the impact on disease transmission, as well as the conclusions of such studies. There is a need for empirical data from, for instance, surveys or discrete choice experiments to support the validity of these models and to guide further research [7,15].

The main goal of this paper is to systematically review and document how and to which extent behavioural immunity has been explored in infectious disease transmission models over the past 5 years. In brief, we aim to investigate to which extent: (i) technological advancements and increased data availability have enriched BCMs, (ii) the literature has coped with the fact that behavioural immunity is often contingent on the disease and not coupled to disease dynamics, (iii) modelling efforts are validated with quantifiable observations and parametrized, (iv) the current models have assessed the importance of social networks in individual decisions, (v) the process of transferring information to behaviour is managed and (vi) irrational behaviour is demonstrated.

In the following sections, we systematically identify and analyse BCMs applied to infectious disease transmission, starting from where a previous review in 2010 left off [7]. These models are categorized in order to distinguish their assumptions, methods, disease and transmission-specific applications and implications. Furthermore, a critical point of view is taken when evaluating these models in terms of their real-life applicability. Current pitfalls and opportunities are identified to support the development of more advanced BCMs in the near future.

## 2. Methods

The strategy and reporting in this review are based on Cochrane guidelines for systematic reviews of intervention [16] and the PRISMA statement [17]. The eligibility criteria and the search query were determined by consensus between all authors, covering expertise in infectious disease modelling and economics.

### 2.1. Search

We searched PubMed and Web of Science (WoS) for records published between January 2010 and December 2015. After discussing and defining the inclusion and exclusion criteria, we obtained our final search query which we used in PubMed on 12 January 2016 and in WoS on 13 January 2016: ‘(behavio* OR decision*) AND (change* OR influence* OR dynamic* OR adapta* OR adapt OR adaptive OR strategic*) AND (infect* OR epidemic OR epidemics OR epidemiology OR epidemiological OR epidemiologic OR pandem* OR outbreak*) AND (disease* OR vaccin*) AND (model OR models OR modelling OR modeling OR simulat* OR transmission*)’.

### 2.2. Selection

In a first step, F.V. screened the results of the search query based on title and abstract in accordance with the following pre-specified eligibility criteria:

*Infectious diseases*. Only records that concern infectious diseases are included in the selection. Infectious diseases are defined using the WHO definition: infectious diseases are caused by pathogenic microorganisms, such as bacteria, viruses, parasites or fungi; the diseases can be spread, directly or indirectly, from one person to another [18].
*Model.* Records should consist of a mathematical model for behavioural change, for infectious disease transmission or a coupled model combining these two.
*Individual behaviour.* Behaviour is considered the consequence of personal and voluntary choices made by an individual, i.e. we exclude studies tackling forced interventions such as school closure or mandatory vaccination, but include government interventions creating awareness, education in prevention, etc.
*External trigger(s).* At least one trigger for modelled individuals to change their behaviour is external and has to be related to infectious disease. We exclude models with exclusively intrinsic triggers from the selection (e.g. an individual's own human immunodeficiency virus (HIV) status).
*Preventive measure.* A preventive measure is central to the analysis (e.g. vaccination or social distancing). The behaviour of the individual is defined by the decision (not) to take preventive measures.
*Humans.* We are interested in diseases in humans and behaviour of humans regarding these diseases, and therefore exclude research on plants, animals, the behaviour of the model itself or the behaviour of governments or institutions.
*Original research.* We exclude review articles, letters, editorials and comments.
*English language.* Excluding articles written in other languages.

In a second step, the remaining articles' full texts were screened to confirm eligibility, independently by F.V. and L.W. Whenever there was doubt about eligibility, agreement was sought through discussion.

### 2.3. Data extraction

Using a common data extraction protocol for each eligible article, F.V. and L.W. independently retrieved from the full text: (i) infectious disease; (ii) disease category (sexually transmitted infection (STI), influenza-like illness (ILI), childhood disease, vector-borne disease (VBD) or other); (iii) prevention measure (vaccination, social distancing etc.); (iv) source of information (global, local or multiple); (v) type of information (prevalence-based, belief-based or multiple); (vi) effect on the model (disease state, model parameters, contact structure or multiple); (vii) disease transmission model description; (viii) BCM description; (ix) whether there was interaction between the behaviour and disease transmission model; (x) whether the analysis incorporated real-life data; and (xi) movement of individuals in the model. When applicable, other interesting information was extracted using free form fields. Again, discrepancies in interpretation were resolved through discussion.

## 3. Results

### 3.1. Search results

**Figure 1.**
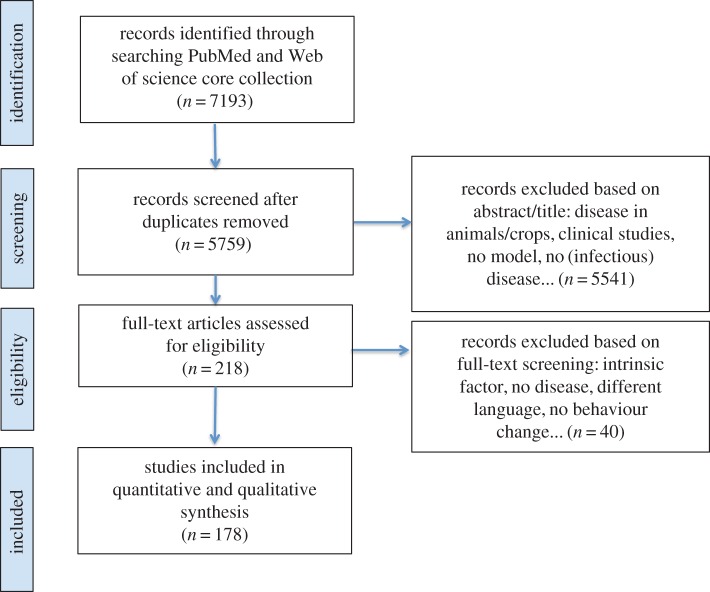
PRISMA flow diagram. (Online version in colour.)

Our search query resulted in 7193 records from Web of Science and PubMed (figure 1). We identified and removed 1434 duplicates, resulting in 5759 unique records that were screened based on title, abstract, keywords and full-text if necessary. Exclusions were mostly related to (i) topic, including the study of non-infectious diseases or infections in animals, plants and crops; (ii) discipline, including microbiological and clinical trial studies, and to a lesser extent to (iii) language and article type. Eventually, 178 articles were included for full-text analysis.

**Figure 2.**
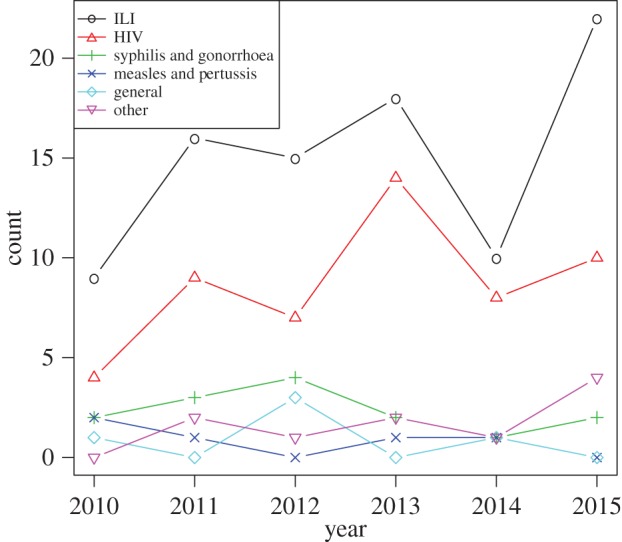
Number of studies over time.

The number of articles matching our eligibility criteria increased from 18 in 2010 to 38 in 2015, but there was a single year downward deviation from the trend in 2014 (with 22 eligible studies; figure 2). Compared with Funk *et al*. [7], we observe a marked increase in BCM publications. Over the 9 year period between 2002 and 2010, Funk *et al.*'s search yielded 27 eligible articles (i.e. about 15% of our yield over 6 years), but their search and selection procedure lacks transparency to compare these results in depth.

Models applied to influenza or ILI stand out, together with ‘general’ models. In the latter category, a hypothetical infectious disease is modelled, without specification of which disease (but often including optimistic statements about the generalizability of the application).

### 3.2. Model structure categories

In table 1, we categorized the studies according to disease, prevention measure (topic) and whether the model is implemented at the population level or at the individual-level (i.e. using an IBM or contact network) to simulate infectious disease transmission. Metapopulation models for disease transmission were also identified and are labelled in bold. Furthermore, the columns indicate at which level the impact of prevention measures is modelled, distinguishing whether behavioural change is implemented through a switch in infectious disease state (e.g. vaccination immunizes previously susceptible persons, and this can be modelled by moving them from the susceptible to the immune state), a change in model parameters (e.g. hygiene measures may be assumed to reduce the effectiveness of transmission) or in social contact structure (e.g. social distancing may be mimicked by a link-breaking or rewiring process between susceptible and infectious individuals in contact networks). Studies can appear in multiple categories, as some have multiple prevention strategies or multiple effects on the disease transmission model. For the transmission model category, we interpreted to which extent heterogeneity is introduced in the model. All references are categorized and represented in a spreadsheet that can be found as electronic supplementary material. The model type is often disease-dependent. For instance, all retrieved models for measles and/or pertussis are population models with vaccination as a preventive measure that affects the disease state in the transmission model. Moreover, the models are often prevention-dependent. We observe that most of the models that use vaccination as a prevention strategy will impact the model through a switch in disease state. For instance, in many compartmental susceptible–infectious–recovered (SIR) disease models, vaccinated individuals move to the R compartment. General models with social distancing as a prevention strategy usually impact the model in terms of a modified contact structure, contingent on the disease transmission model. Whereas for influenza applications, this only applies for one out of seven references.

**Table 1.** Model structure categories. References in bold represent metapopulation models. References with author names in italics represent references that use empirical data for parametrization and/or validation. PrEP, pre-exposure prohylaxis. References in category ‘Other’ specify a disease other than the above. Hygiene measures include face-mask use, increased hand washing, etc. For more information, see online appendix.

		population level		network/IBM		
disease	prevention measure	infectious disease state	model parameters	infectious disease state	model parameters	contact structure
influenza/ILI	vaccination	Bhattacharyya & Bauch [19], Liu *et al*. [20], Breban [21], Laguzet & Turinici [22], *Shim et al.* [23]*, Cohen et al.* [24]*, Xia* & *Liu* [25]	Vardavas *et al*. [26]	Liu *et al*. [20], Cardillo *et al*. [27], Wu & Zhang [28], Zhang [29], Han & Sun [30], Fukuda *et al*. [31], Cornforth *et al*. [32], Fu *et al*. [33], Loganathan *et al*. [34], Wells & Bauch [35], Zhang *et al*. [36], Wells *et al*. [37], Xia & Liu [38], Fukuda *et al*. [39], Liao & You [40], *Andrews & Bauch* [41]	Marathe *et al*. [42], Mei *et al*. [43], Zhang *et al*. [44], *Karimi et al.* [45]	—
	Social Distancing	Poletti *et al*. [46], Mummert & Weiss [47], *Zhong et al.* [48]	Larson & Nigmatulina [49], Wang [50], Greer [51], Morin *et al*. [52], Liu *et al*. [53], *Poletti et al.* [54]*, He et al.* [55]*, Springborn et al.* [56]	—	Mei *et al*. [43], Karimi *et al*. [45], Barrett *et al*. [57,58], Wang *et al*. [59], ***Bayham et al.*** [60]	Chen *et al*. [61]
	PrEP antivirals	—	—	Liao & You [40]	Liao & You [40], Marathe *et al*. [42], Mei *et al*. [43], Barrett *et al*. [58], Chen *et al*. [61], Mao & Bian [62], Mao & Yang [63], *Andrews & Bauch* [41]	—
	hygiene measures	—	Larson & Nigmatulina [49], Parikh *et al*. [64], *Poletti et al.* [54]	—	Mei *et al*. [43], Wang *et al*. [59], Parikh *et al*. [64]	—
	other and general	—	*Xiao et al.* [65]*, Pawelek et al.* [66]	Liao *et al*. [67], *Collinson et al.* [68]	Zhang *et al*. [69], *Fierro & Liccardo* [70]	—
HIV	condom use	—	*Nyabadza et al.* [71]*, Johnson et al.* [72]	—	Vieira *et al*. [73]	—
	reduce sexual risk	—	—	Alimadad *et al*. [74]	Tully *et al*. [75,76]	—
	social distancing	—	Nyabadza *et al*. [71,77], Reniers & Armbruster [78], Reluga & Li [79], *Viljoen et al.* [80]	—	Tully *et al*. [75]	—
	other and general	Kassa & Ouhinou [81,82]	—	—	Marshall *et al*. [83]	—
syphilis and gonorrhoea	condom use	—	Morin *et al*. [84]	—	*Gray et al.* [85]	—
	vaccination	—	Milner & Zhao [86]	—	—	—
	reduce sexual risk	—	Milner & Zhao [86]	—	—	—
	social distancing	—	Aadland *et al*. [87]	—	*Gray et al.* [85]	*Althouse & Hébert-Dufresne* [88]
measles and pertussis	vaccination	Bhattacharyya & Bauch [89], Shim *et al*. [90], *Bauch & Bhattacharyya* [91]*, d'Onofrio et al.* [92]*, Oraby et al.* [93]	—	—	—	—
other	social distancing	Perra *et al*. [94], Sharma & Misra [95], Greenhalgh *et al*. [96]	Cooper *et al*. [97], Del Valle *et al*. [98], Wang *et al*. [99]	—	*Fast et al.* [100]	*Robinson et al.* [14]
	vaccination	Sharma & Misra [95], Sykes & Rychtar [101]	—	—	—	—
	other and general	Misra *et al*. [102]	Cooper *et al*. [97]	Williams *et al*. [103]	—	—
general models	social distancing	Funk *et al*. [104], Misra *et al*. [105], Samanta *et al*. [106], Zuo & Liu [107], Misra *et al*. [108], Wang *et al*. [109]	Reluga [110], Brauer [111], Fenichel *et al*. [112], Buonomo *et al*. [113], Xiao *et al*. [114], Liu [115], Wang *et al*. [116], Li *et al*. [117], Morin *et al*. [118], Li *et al*. [119], Liu *et al*. [120], Lubuma & Terefe [121]	Sahneh & Scoglio [122]	Wu *et al*. [123], Wang *et al*. [124], Cao [125], Zhang *et al*. [126], *Schumm et al.* [127], **Meloni *et al*.** [128], **Sun** ***et al*.** [129], **Bichara** ***et al*.** [130], **Liu & Zheng** [131]	Van Segbroeck *et al*. [12], Shaw & Schwartz [132], Jolad *et al*. [133], Maharaj & Kleczkowski [134], Rogers *et al*. [135], Valdez *et al*. [136], Zhang *et al*. [137], Juher *et al*. [138], Liu *et al*. [139], Noble *et al*. [140], Valdez *et al*. [141], Tunc & Shaw [142], Dong *et al*. [143], Szabo-Solticzky *et al*. [144], **Nicolaides *et al*.** [145], *Maharaj et al.* [146]
	vaccination	Bauch *et al*. [147], d'Onofrio & Manfredi [148], Buonomo *et al*. [149], d'Onofrio *et al*. [150], Reluga & Galvani [151], Wu *et al*. [152], Buonomo *et al*. [153], Xu & Cressman [154], Bhattacharyya *et al*. [155], Oraby & Bauch [156], Voinson *et al*. [157]	Barbagallo & Cojocaru [10]	Mbah *et al*. [13], Dong *et al*. [143], Schimit & Monteiro [158], Wells *et al*. [159], Morsky & Bauch [160], Ruan *et al*. [161], Campbell & Salathe [162], Wu *et al*. [163], Cai *et al*. [164], Wu *et al*. [165], Han *et al*. [166], Helbing *et al*. [167], Liang & Juang [168]	Zhang *et al*. [169]	—
	hygiene measures	Funk *et al*. [104]	—	—	—	—
	other and general	Joshi *et al*. [170]	Reluga [11], Liu & Stechlinski [171], Samanta & Chattopadhyay [172], Sega *et al*. [173]	Hatzopoulos *et al*. [174], Sahneh & Scoglio [175], Sahneh *et al*. [176], Miller [177], Yuan *et al*. [178], Goyal & Vigier [179], Guo *et al*. [180], Juher *et al*. [181], Liu *et al*. [182], *Chen et al.* [183]	Goyal & Vigier [179], Kitchovitch and Lio [184,185], Ni *et al*. [186], Rutherford *et al*. [187], Shang [188,189]	—

### 3.3. Prevention measures

Most of the eligible articles use models with vaccination or social distancing as a prevention measure, though other strategies have been considered. The choice of prevention measure naturally depends on the disease under study. For instance, the discovery and implementation of antivirals as a prophylactic for influenza and HIV has resulted in the publication of models with pre-exposure antiviral use as individual behaviour. A minority of models does not specify the preventive action taken by individuals. When an effect on the contact rate was mentioned, we assumed that the preventive action was social distancing. It appears some authors use the term ‘social distancing’ as a synonym for all non-pharmaceutical interventions (NPIs) [11]. In this review, social distancing is interpreted as reducing physical (or sexual) contacts between individuals and their environment.

### 3.4. Diseases

In table 1, we classified the records based on four specific disease categories, one category for general models (not specifying a disease) and one category for other diseases. Most models retrieved were on influenza or influenza-like illness (ILI) and HIV. Other frequent diseases studied with BCMs are ‘measles & pertussis’ and ‘syphilis & gonorrhoea’. Historically, perceptions of high risks, associated with measles and pertussis vaccination, have adversely affected the uptake of these vaccines. As a result, these are topical applications for transmission models incorporating behavioural changes, as discussed in [19]. The literature on measles is becoming more diverse as VRSE perceptions evolve; Bhattacharyya & Bauch [89] describe a model in which parents delay vaccinating their children as a result of an exogenous vaccine scare, whereas the same authors use social networks of imitation behaviour for VRSE perception spread in response to a vaccine scare [91], and d'Onofrio *et al*. [92] introduce public interventions in their model to increase vaccine uptake. Diseases in the ‘other’ category are: SARS, smallpox-like disease, malaria, hepatitis B, Ebola, pneumococcus, pneumonic plague, toxoplasmosis and cholera. General models do not explicitly specify a disease, often assuming general applicability. As noted earlier, models tend to be disease-specific. In the case of influenza or influenza-like illness, some models look at seasonal changes in behaviour with backward looking individuals evaluating the success of their (vaccination or social distancing) strategy during previous season(s) [20,27–31,69]. HIV BCMs are often coupled with a public health information/education campaign aimed at evaluating public health measures to control epidemic spread or to study the cost-effectiveness of these control measures [71,72,77,81,83]. An example of a more advanced, game-theoretic model is the model by Tully *et al*. [75]. They use an agent-based model (ABM) for the spread of risk perception, sexual behaviour and HIV transmission in the context of individual sexual encounters evaluating the behaviour of (potential) partners.

### 3.5. Emergence-driven research

Between 2010 and 2015, much research has been emergence-driven. That is, the research field responds by focusing on diseases that are of major interest because of a change in the threat they present to public health. The influenza A/H1N1 pandemic of 2009 has largely influenced the development of BCMs for influenza. For example, Poletti *et al*. [54] use the influenza A/H1N1 pandemic of 2009/2010 to parametrize an influenza transmission model with behavioural changes focusing on the spread of risk perceptions in the population. In addition, a model on Ebola virus disease (EVD) was published in 2015 in response to the epidemic outbreak in Liberia [100]. The authors use WHO and CDC data to parametrize the model suggested in an attempt to mimic disease transmission and to identify behavioural changes as drivers of the disease dynamics. Note that, in the current review, we relate ‘emergence’ not only to disease emergence, but also the emergence of a vaccine scare (such as observed with measles–mumps–rubella (MMR) vaccination and pertussis whole-cell vaccination [91]) or the emergence of new therapies for endemic diseases (such as the development of a multi-season influenza vaccine [26]).

### 3.6. Disease transmission models

We identify three major categories of models: population-level models, metapopulation and individual-level models. Population-level models traditionally formulate compartments according to health state (e.g. susceptible, infectious and recovered) and simulate transitions between the compartments over time using population averages. These models are often based on the mass-action principle to designate the transmission probability. Each individual has an equal probability of contracting disease given the disease state levels in the population. Metapopulation models split the population into different subpopulations with their own (spatial) general characteristics and disease-related parameters. The individual-level category consists of network models and IBMs. Network models represent disease transmission on a network where nodes (individuals) are connected to each other using links. This allows to model individuals with different degrees, representing how many links a node has (i.e. number of neighbours/direct contacts). IBMs or ABMs typically incorporate more heterogeneity and stochasticity on individuals' characteristics such as spatial location, age, gender, sexual orientation, etc. The model selection depends on disease characteristics, data availability, modelling purpose (i.e. what outcome figures are you interested in?), computational resources, etc.

Individual-level models are gaining interest in the BCM literature since they can introduce heterogeneity in behaviours, tackle clustering of vaccine sentiments and look at stochastic and local outbreaks of infectious diseases with a high vaccination coverage (e.g. measles). Moreover, given an underlying contact structure, individual-level models are well suited to model social distancing behaviour in terms of reduced contacts as a prevention strategy. Remarkably, for measles and pertussis we found deterministic models only, despite the widely acknowledged stochastic nature of outbreaks in highly vaccinated populations. Note that, in table 1, we also made a distinction between individual-level and population-level models in the category ‘disease transmission model’. Metapopulation models are displayed in bold.

### 3.7. Information gathering

In order for individuals to change their behaviour in relation to prevention measures, they require disease-related information. As defined in the eligibility criteria, we only included papers in which this information is external to the individual. Examples of disease-related, external information include: news broadcasts on a disease outbreak or rumours among friends and family about VRSEs or vaccine-preventable disease. Funk *et al*. [7] proposed a classification based on type and source of information, distinguishing global and local information as source and prevalence-based and belief-based information as type of information. Global information is defined as information available to all individuals in the population, for example, TV stations and public health campaigns. Local information is information individuals gather from their direct contacts or neighbourhood. Examples are rumours from neighbours or infective individuals in their close contacts. Prevalence-based information is defined as ‘directly relating to disease prevalence’, whereas belief-based information is ‘not directly relating to disease prevalence’. Belief-based information can therefore have its own dynamics, to some extent independent of the disease dynamics. For example, rumours can inflate the perception of disease prevalence, even if the true prevalence is low. In table 2, we classify the studies we identified in a matrix, using the same definitions.

**Table 2.** Information gathering.

	type of information		
source of information	belief-based	prevalence-based	multiple
local	Barbagallo & Cojocaru [10], Mbah *et al*. [13], Liu *et al*. [20], Cardillo *et al*. [27], Wu & Zhang [28], Zhang *et al*. [69], Zhang [29], Han & Sun [30], Fukuda *et al*. [31], Xia & Liu [25,38], Wang *et al*. [59], Alimadad *et al*. [74], Tully *et al*. [76], Funk *et al*. [104]	Van Segbroeck *et al*. [12], Fu *et al*. [33], Fukuda *et al*. [39], Reniers & Armbruster [78], Althouse & Hébert-Dufresne [88], Sahneh & Scoglio [122,175], Cao [125], Zhang *et al*. [126], Schumm *et al*. [127], Shaw & Schwartz [132], Rogers *et al*. [135], Valdez *et al*. [136], Juher *et al*. [138], Noble *et al*. [140], Valdez *et al*. [141], Tunc & Shaw [142], Dong *et al*. [143], Szabo-Solticzky *et al*. [144], Maharaj *et al*. [146], Wells *et al*. [159], Morsky & Bauch [160], Ruan *et al*. [161], Cai *et al*. [164], Wu *et al*. [165], Han *et al*. [166], Helbing *et al*. [167], Sahneh *et al*. [176], Juher *et al*. [181], Liu *et al*. [182]	Zhang *et al*. [36], Wells *et al*. [37], Andrews & Bauch [41], Mao & Bian [62], Mao & Yang [63], Wu *et al*. [163], Miller [177], Guo *et al*. [180], Shang [189]
global	Bhattacharyya & Bauch [19,89], Bauch & Bhattacharyya [91], Johnson *et al*. [72], Marshall *et al*. [83], Laguzet & Turinici [22], Shim *et al*. [23], Cornforth *et al*. [32], Karimi *et al*. [45], Poletti *et al*. [46], He *et al*. [55], Parikh *et al*. [64], Vieira *et al*. [73], Milner & Zhao [86], Shim *et al*. [90], Oraby *et al*. [93], Cooper *et al*. [97], Fast *et al*. [100], Sykes & Rychtar [101], Misra *et al*. [102], Li *et al*. [117], Voinson *et al*. [157], Zhang *et al*. [169], Joshi *et al*. [170], Durham & Casman [3], Mannberg [190]	Reluga [11], Nyabadza *et al*. [71,77], Kassa & Ouhinou [81], Zhang *et al*. [44], Mummert & Weiss [47], Zhong *et al*. [48], Wang [50], Greer [51], Morin *et al*. [52], Liu *et al*. [53], Poletti *et al*. [54], Barrett *et al*. [57], Chen *et al*. [61], Xiao *et al*. [65], Pawelek *et al*. [66], Collinson *et al*. [68], Reluga & Li [79], Viljoen *et al*. [80], Morin *et al*. [84], Aadland *et al*. [87], Sharma & Misra [95], Greenhalgh *et al*. [96], Del Valle *et al*. [98], Wang *et al*. [99], Misra *et al*. [105], Samanta *et al*. [106], Zuo & Liu [107], Misra *et al*. [108], Wang *et al*. [109], Reluga [110], Fenichel *et al*. [112], Buonomo *et al*. [113], Xiao *et al*. [114], Liu [115], Wang *et al*. [116], Morin *et al*. [118], Li *et al*. [119], Liu *et al*. [120], Lubuma & Terefe [121], Wang *et al*. [124], Meloni *et al*. [128], Sun *et al*. [129], Bichara *et al*. [130], Liu & Zheng [131], Jolad *et al*. [133], Zhang *et al*. [137], Liu *et al*. [139], d'Onofrio & Manfredi [148], Buonomo *et al*. [149], d'Onofrio *et al*. [150], Reluga & Galvani [151], Wu *et al*. [152], Xu & Cressman [154], Liu & Stechlinski [171], Samanta & Chattopadhyay [172], Yuan *et al*. [178], Goyal & Vigier [179], Chen *et al*. [183]	Breban [21], Bayham *et al*. [60], Liao *et al*. [67], Bauch *et al*. [147], Buonomo *et al*. [153], Bhattacharyya *et al*. [155], Oraby & Bauch [156], Sega *et al*. [173]
multiple	d'Onofrio *et al*. [92], Cohen *et al*. [24], Loganathan *et al*. [34], Nicolaides *et al*. [145], Campbell & Salathe [162], Ni *et al*. [186]	Vardavas *et al*. [26], Marathe *et al*. [42], Larson & Nigmatulina [49], Barrett *et al*. [58], Kassa & Ouhinou [82], Maharaj & Kleczkowski [134], Schimit & Monteiro [158], Kitchovitch & Lio [184,185], Shang [188]	Tully *et al*. [75], Wells *et al*. [37], Liao & You [40], Mei *et al*. [43], Fierro & Liccardo [70], Perra *et al*. [94], Wu *et al*. [123], Liang & Juang [168], Hatzopoulos *et al*. [174]

We observe that most BCMs are using information that is globally available and prevalence-based. These models are frequently game-theoretic (or pay-off maximizing) behavioural change frameworks coupled with disease transmission models at the population level. Studies that met our eligibility criteria, but are unclear about the information individuals use [14,56,85,103,111,187] were excluded from figure 2. Given the increasing individual heterogeneity in disease transmission models, it is becoming more interesting to incorporate local information in BCMs. In network models and IBMs, one could for instance model the local spread of information through direct contacts with crucial implications in terms of clustering of both disease prevalence and opinions [186].

In addition, we observe that more articles are using multiple information types and/or sources, making individual behaviour more realistic. For instance, Barrett *et al*. [58] constructed a model where ‘individual behaviour is triggered by the prevalence level of the virus in the overall society (global prevalence) as well as within one's own demographic class (local prevalence)’. Highly relevant are articles introducing both multiple sources and multiple types of information such as the *bij* model, Liang & Juang [168], which introduces different forms of information in the individual's risk perception of an epidemic, embodying all four information categories.

### 3.8. How is the transfer from information to behaviour managed?

Based on full-text analysis, we extracted how individuals were modelled to translate the information they receive into behavioural change. Traditionally, behaviour formation models were composed of a game-theoretic framework in which individuals have perfect information on disease-related data and prevention effectiveness. Individuals are then assumed to use this information in a utility-maximizing game by comparing the expected costs of infection with the expected costs of the prevention measure. However, more advanced and different BCMs have been developed since. We identified five distinct categories for characterizing the decision-making process of individuals, listed in §§3.8.1–3.8.5. (see also electronic supplementary material, appendix). Some referenced papers contain multiple BCMs.

#### 3.8.1. Exogenous behaviour formation (16/178)

We retrieved 16 papers [14,45,51,56,64,72,73,83–85,97,98,103,111,170,187] describing BCMs in which there is no two-way interaction with a disease transmission model. Morin *et al*. [84] provide an example of such a model by assessing the impact of policies encouraging condom use, on gonorrhoea transmission dynamics; i.e. behaviour (condom use) is parametrized based on different empirical studies and model projections are made to estimate consequential disease transmission and model equilibria. Similarly, Brauer [111] assessed disease model implications of a constant fractional reduction in the number of contacts. A third example is the model by Joshi *et al*. [170] where a time-dependent education function moves susceptible individuals into lower susceptibility classes with lower transmission rates, independent of disease dynamics. These models are relatively rare and most often focus on policy implementations and short-term effects of behaviour on disease transmission.

#### 3.8.2. Information threshold (29/178)

We retrieved 29 BCMs in which behaviour change is modelled conditional on exceeding a predefined information threshold [12,42,57,58,61–63,70,78,81,88,114,127,132,133,135,136,138–144,162,163,180–182]. The information the individual assesses can be obtained in a direct way (e.g. through prevalence in neighbours) or in an indirect way (e.g. through rumours or opinions). These models do not elaborate on how behaviour is rationally determined or influenced by relevant factors. Instead, behaviour formation is a result of a predefined threshold function. Examples include switching to social distancing when the number of infectives exceeds a threshold [114], social distancing by rewiring once a non-infected node connects to an infected node [132], and—as in Wu *et al*. [163]—to have an individual's vaccination decision exercised through a risk function exceeding a threshold, which in turn depends on the number of infected neighbours. Mao & Yang [63] used an individual risk function incorporating the proportion of ‘adopters’ among contacts, the perceived pressure of ‘adoption’ and the proportion of infective neighbours. Again, once the risk function exceeds a threshold, individuals adopt preventive behaviour, which in this case consisted of taking prophylactic antivirals.

#### 3.8.3. Information as dynamic parameter (76/178)

The largest category embodies 76 references managing information as a dynamic parameter [3,25,34,40,43,47–50,53,55,60,61,65–68,70,71,74,77,80,82,86,92,94–96,99,100,102,104–109,113,115–117,119–126,128–131,133,134,137,148,149,153,157,161,165,166,168,171–178,184–186,188,189]. In this category, instead of a threshold, the information is a continuous input in the decision-making process of individuals. At the population level, we can characterize these BCMs as information driving the flow in and out the prevention taking compartment. Two subcategories can be distinguished: models with a direct relation between infectious disease parameters and behaviour formation (i.e. behaviour changes vis-à-vis disease dynamics), and models with an indirect relation, through an information spread medium. For the former subcategory, the behaviour or decision-making process is predefined as a functional relation depending on disease transmission parameters. The functional form does not need to be linear. Some examples are vaccination coverage as a positive decreasing function of perceived risk of VRSE [148], the percentage of the susceptible population engaging in avoidance actions increases as the disease becomes more prevalent [48] and a model where the effective contact rate reduces with the number of infectives [119].

The latter subcategory requires a third-party spreading the information for individuals to receive. For instance through mass media, neighbours, formation of opinions in the population, etc. A multitude of these models introduce an ‘aware’ compartment in the model where aware and unaware individuals are assigned distinct disease transmission parameters such that aware individuals have lower susceptibility of acquiring infection. See for example Funk *et al*. [104], in which a rate introduces people in an ‘aware’ class after which the awareness spreads through the population, coupling disease transmission with a BCM. Interestingly, some models introduce information spread models with characteristics from disease transmission models where individuals are, for example, susceptible to or infected with disease-related information. Misra *et al*. [105] use a model with media coverage creating awareness in the population, also introducing an ‘aware’ compartment in a population model. Social impact is introduced in a model by Ni *et al*. [186], where they use a variety of complex networks for the spread of opinions driving the individual probability of prevention behaviour. The use of a network is convenient to model these dynamics as they allow clustering of, for instance, vaccine-related sentiments in the population. Most often these models assign additional characteristics to nodes (which represent individuals), apart from disease state. An example could be that a node is assigned a disease state and an opinion which is either provaccination or contravaccination. When simulating the disease and behaviour dynamics in this network, when nodes interact, transmission of both disease and opinions can occur. Such that if a provaccine node is surrounded by many vaccine sceptics, it might change its opinion towards the opinions of its links (i.e. neighbours) and as a result this will influence the individual's probability of taking vaccination as a prevention measure.

#### 3.8.4. An economic objective function (37/178)

This ‘economic’ class of BCMs is also quite common with 37 articles being retrieved [10,11,13,19,21–24,26,32,35,41,52,59,75,76,79,87,90,101,110,112,118,128,145–147,151,155,157,158,160,167,169,179,183,190]. This approach assumes individuals take their prevention decision based on an objective function, which they attempt to optimize (i.e. by maximizing benefits and/or minimizing costs). Game theory grounded models form an integral part of this category. By way of example, one can assume that individuals have knowledge about both the disease and their options for prevention and make rational decisions based on this knowledge. People accordingly possess a (perceived) cost of infection (*c*_i_) and a (perceived) cost of the prevention measure (*c*_p_), which can, for instance, be assumed to be 100% effective. Another important input in people's decision-making, their probability of infection (*λ*) can be assumed to be dependent on disease prevalence, which evolves over time. For instance, one can define this using an SIR model under the mass action principle as the force of infection, i.e. *λ* = *βI*, where *β* is the per-contact transmission rate, and *I* is the fraction of infectives in the population. This way the behavioural change framework can be coupled to the disease dynamics. The individual makes the following trade-off, with *P*, the choice of taking the prevention measure3.1
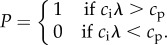


In a study by Bhattacharyya & Bauch [19], individuals take their vaccination decision based on the perceived vaccination cost in the context of the 2009 A/H1N1 influenza pandemic. Their BCM model exhibits a ‘wait and see’ Nash equilibrium where individuals incorporate the concept of herd immunity in their prevention behaviour, resulting in free-riding represented by a ‘delayer’ strategy. The model developed by Morin *et al*. [52] embodies individuals' behaviour by the maximization of expected utility determined by adapting the contact level (i.e. social distancing). Aadland *et al*. [87] introduce a BCM maximizing an individual's expected lifetime utility by choosing the number of sexual partners, hereby explaining the re-emergence of syphilis.

#### 3.8.5. An economic objective function with social learning/imitation (26/178)

**Figure 3.**
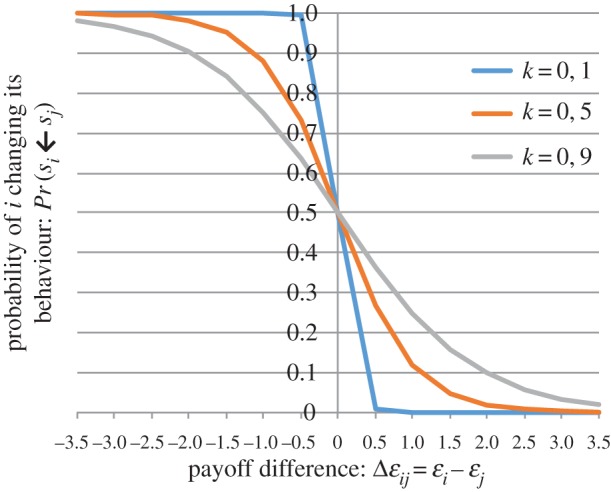
Fermi function for different values of *κ.*

We retrieved 26 papers [13,20,27–31,33,37–39,44,46,54,69,89,91,93,137,143,150,152,154,156,159,164] describing a BCM with an objective function with imitation. It is recognized that some social or peer influence should be incorporated in the decision-making process of the individuals (see also models with information as a dynamic parameter). As a response to this concern, the (rational) ‘game-theoretic’ model has been adapted to include social influence or imitation behaviour. In these models, it is assumed that people compare their own prevention-related behaviour with that of other individuals in society. Through comparison, individuals learn whether their own behaviour is optimal and, to which extent they should adapt it. Typically, a sampling rate is assumed for individuals sampling other individuals from the population. After sampling an individual from the population, the trade-off is compared and people switch strategies with a probability as a function of the pay-off difference. Often, a Fermi-like function is used, guiding the adoption to the better strategy depending on the magnitude of the pay-off difference. Other switching functions/strategies are used, but naturally, the larger the beneficial pay-off difference, the higher the probability of switching your behaviour. An example of a Fermi function, taken from [31] is given in this section. If we represent the pay-off of the strategies of individual *i* (with strategy *s_i_*) and individual *j* (with strategy *s_j_*) as *ɛ_i_* and *ɛ_j_* respectively, and the pay-off difference is defined by Δ*ɛ_ij_* = *ɛ_i_* − *ɛ_j_*. Then, the probability of individual *i* switching to the strategy of individual *j* is3.2
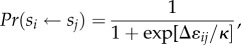
where *κ* denotes the selection pressure representing the sensitivity of individuals to switch strategies in response to a pay-off difference [31]. Parameter *κ* can be interpreted as expressing ‘stickiness’ in behaviour. Figure 3 indicates that individuals are very responsive even to small differences in the pay-off when *κ* is low, and that for large values of *κ* (e.g. 0,9) their behaviour becomes ‘sticky’. Sticky, in the sense that they need to observe a very large pay-off difference before they opt to change. For intermediate values of *κ*, people have sticky behaviour but when the potential benefit in the pay-off is large enough, people switch to the strategy of individual *j*. If the behaviour is not assumed to be very sticky, then it could be that individual *i* still adopts the strategy of individual *j* even if the pay-off of strategy *j* is worse. The underlying assumption is here that for some individuals peer influence and social conforming behaviour is—to a certain extent—more important than pay-off maximization. Note that in the majority of these models, assumptions rather than real-life observations guide the choice and distribution of the ‘stickiness’ parameter *κ*.

### 3.9. Model parametrization and validation

One may question how well BCMs approach reality, as there is a paucity of empirical data on behavioural responses to disease-related information informing these models. We examined whether and how data were used to parametrize BCMs, and to which extent these data support the underlying theoretical model. Moreover, we critically assessed model parametrization, distinguishing data-driven from assumption-driven parametrization, for the disease model, the BCM and the complete integrated model. A first, striking observation is that most models are solely theoretical because they are constructed independently from empirical observations. Often a stability analysis is performed, and equilibria are obtained in order to grasp the dynamics of the model in the absence of parameter values. Others perform numerical simulations with either assumptions on parameters or referring to other studies supporting their choice of parameters. Less than 20% of the studies has (partially) fitted or validated their model to behavioural and/or disease transmission data. Retrospective studies on disease emergence are particularly useful when real-time data on behavioural change and disease transmission during an outbreak are available over a sufficiently long time. Social media data and other electronic sources of information are also increasingly used, thus creating opportunities for ‘big data’ collection on disease transmission, behaviour formation and spatial location [25,60,66]. Next, we briefly describe studies constructing their models using observational data, i.e. studies not exclusively making assumptions or taking parameters from literature.

To underpin BCMs, participatory experiments have been performed to capture social distancing. Maharaj *et al*. [146] and Chen *et al*. [183] collected data through a game in which participants trade-off social contacts versus their risk of infection. Such data can be used to parametrize game-theoretic models of social distancing and adaptive networks with link deletion. In addition, survey data have been used to assess behavioural change. Zhong *et al*. [48] used survey (Public Risk Communication Survey, 2009) data to parametrize their BCM. Robinson *et al*. [14] surveyed sexual attitudes and lifestyle to build a sexual contact network. The IBM in Gray *et al*. [85] for syphilis transmission was also informed with survey data on sexual behaviour. Additionally, disease transmission parameters were calibrated from syphilis diagnosis among gay men in Victoria, Australia. A survey on altruism and self-interest was conducted by Shim *et al*. [23] to calibrate the behavioural change parameters regarding influenza vaccination. In Schumm *et al*. [127], the BCM is represented by a dynamic social contact network with social distancing, constructed from a survey and census data. Cohen *et al*. [24] surveyed a convenience sample of students about their risk perceptions for influenza A/H1N1 to estimate the utility values of different behaviours. The study by Fierro & Liccardo [70], used data on awareness and concern about the risk of contagion to populate their model on A/H1N1 influenza transmission with behavioural parameters. Moreover, they also validated their output through comparisons with Italian influenza surveillance data from 2009. The health belief model (HBM) [191] is frequently used to retrieve prevention behaviour and parametrize BCMs. The parameters in the HBM in Durham & Casman [3] were calibrated, using survey data on perceived severity and susceptibility during the 2003 SARS outbreak in Hong Kong. Karimi *et al*. also use the HBM for their ABM on influenza in 2015 [45]. For validation, the authors compare their model output with similar influenza ABMs in the literature. Another model tackling the influenza A/H1N1 pandemic in 2009 is the model by Bayham *et al*. [60], who used data from the American time-use survey and the National Health and Activity Patterns Survey (NHAPS). Moreover, Google Trends data are represented as a proxy for subjective risk perception and weather data are used to control for the effects of extreme weather phenomena. Xia *et al*. [25] constructed a social network using data of an online Facebook-like community to construct a BCM for disease and vaccine awareness on the 2009 influenza A/H1N1 pandemic in Hong Kong. The same pandemic has inspired Springborn *et al*. to use home television viewing as a proxy for social distancing [56]. Pawelek *et al*. [66] used Twitter data of self-reporting for awareness spread and ILI surveillance data (UK Health Protection Agency) of the 2009 A/H1N1 influenza pandemic for disease transmission. In addition, Collinson *et al*. [68] constructed a model on influenza A/H1N1, incorporating mass media report data from the Global Public Health Intelligence Network.

Incidence and outbreak data have been useful to inform the disease dynamics parallel with BCMs. For the 2009 influenza pandemic, Zhong *et al*. [48] parametrized their transmission model with outbreak data from Arizona and Xiao *et al*. [65] estimated parameters using outbreak data (laboratory-confirmed cases) from Shaanxi province in China. Schumm *et al*. [127] focused on observational census and survey data from rural areas. Andrews & Bauch [41] calibrated both disease and behaviour parameters to vaccine coverage and disease incidence data. Althouse & Hébert-Dufresne [88] used surveillance-based incidence rates for syphilis and gonorrhoea from 1941 to 2002. Gray *et al*. [85] calibrated disease transmission parameters from data on syphilis diagnosis among men who have sex with men in Victoria, Australia. An HIV transmission model including adaptive condom use and sexual partnerships in South Africa is fitted to HIV prevalence data in Nyabadza *et al*. [71]. The publication makes projections for disease dynamics when scaling up condom use and reducing the number of sexual partners stepwise with 10%. Behavioural change parameters are not calibrated in this publication. The HIV model of Viljoen *et al*. [80] is fitted to prevalence data in South Africa and Botswana to look at the effect of awareness on disease spread.

BCMs on vaccination dynamics have also been supported by real-life observations. Bauch & Bhattacharyya [91] informed model parameters with historical vaccine coverage and disease incidence data from two vaccine scares (MMR and whole-cell pertussis). The behavioural change framework introduced in the model has a game-theoretic foundation with inclusion of imitation. Likewise, a model for the dynamics of vaccine uptake with a public intervention was proposed by d'Onofrio *et al*. [92]. Pertussis vaccination uptake and disease dynamics data for the UK are used to fit the model by Oraby *et al*. [93], which focuses on the inclusion of injunctive social norms in the context of vaccinations for paediatric infectious diseases. The model is validated comparing the model prediction with observed vaccination uptake data during both the UK vaccine-scare period and high coverage period.

Model fitting has been performed through maximum-likelihood and least-squares methods [3,55]. Poletti *et al*. [54] use ILI incidence data in Italy to calibrate the disease dynamics in their game-theoretic model using least-squares. In addition, data on antiviral drug purchase were used to calibrate the model. In [100], a model of social mobilization is fitted to weekly case counts from CDC and WHO for EVD in Lofa County, Liberia. He *et al*. [55] investigated three possible explanations for multiple waves of the 1918 influenza pandemic, with one consisting of human behaviour responses. Three proposed models are fitted to historical mortality data using maximum-likelihood in order to determine the extent they can justify the observed disease dynamics. Johnson *et al*. [72] used prevalence data, antenatal clinic surveys and household surveys for parametrization in order to determine the effects of increased condom use and antivirals on disease dynamics. They calibrated both disease and behaviour parameters to age-specific data using a Bayesian approach for two distinct models.

## 4. Discussion

### 4.1. What are current behavioural change models capturing?

It is intuitively logical to include human behaviour in mathematical models for the spread of infectious diseases. After all, disease dynamics are, in essence, dependent on human behaviour dynamics: people interact and take preventive measures on a regularly basis. Because there is much heterogeneity in the ways in which behaviour is included and parametrized in BCMs, it seems the real question is: ‘How should behaviour be taken into account?’ We found that model output may depend on the model specification, to the extent that the selection and development of a model leads in a predictive way towards a predefined conclusion. That is, it seems many of these models serve to justify a theory. For instance, in many pure game-theoretic models, free-rider behaviour emerges resulting in suboptimal vaccination coverage levels, whereas in models including imitation behaviour, the results are often ambiguous. Validation of models with real-life observations is desperately needed to specify an appropriate model, conditional on disease characteristics. Note that model selection implicitly determines the characterization of individuals in the population; models with an economic objective function often assume rational decision-makers, whereas models with imitation or information spread introduce some ‘irrational’ behaviour such as peer influence and social responsibility.

Primary sources such as surveys are needed to empirically underpin the foundations of the models used. The study of Skea *et al*. [192] on MMR vaccination decisions uses an online chat forum to assess vaccination sentiments and the importance of social responsibility in the parental decision process. The authors find that: ‘participants expressed a desire to both (i) protect their own child and (ii) help protect others by contributing to herd immunity’ [192]. This finding suggests that people are not purely self-interested and herd immunity is not taken as a means to opt for free-riding, on the contrary, establishing herd immunity is seen as an additional incentive, protecting others. A similar conclusion can be drawn from Vietri *et al*. [9], who tested whether college students consider either free-riding or altruistic motives to decide on (not) receiving vaccinations. They find that individuals both incorporate their own risk of infection and altruistic motives in their decision of whether or not to vaccinate. Determann *et al*. [193] suggest that these behaviours—and as a result the decision-making process—are country-dependent. They find that focus group participants tend to ‘base their vaccination decision on the trade-off between perceived benefits and barriers of the vaccine…’. Although, in their vaccination strategy, Swedish participants also incorporate: following the rules, doing the right thing, solidarity with other citizens and social influences. The latter drivers are less important in Dutch and Polish participants. This implies that studies may have to be diversified by country-specific characteristics to tackle the inhabitant's behaviour. Dorell *et al*. [194] conclude that one of the most important factors for vaccination is the healthcare provider's recommendation, which is a determinant that is not included in any of the approaches in the models we found in this extensive review.

In general, there is a need for empirical research to underpin the development of valid models approximating real-life behaviour and disease transmission. Some attempts for recent BCMs illustrate the difficulty of finding suitable observational data. For instance, Springborn *et al*. [56] used television viewing habits (average viewing time) as a proxy for social distancing, although this proxy is far removed from a direct estimation of social distancing in an outbreak situation. More promising sources of information include: survey data using, for instance, the HBM framework (also see [191,195,196]) or time-use surveys [3,14,23,24,45,48,60,72,85,127,183] or digital sources such as social media [25,60,66,146,197]. Real-life data collection during the influenza A/H1N1 pandemic in 2009 has been a milestone for the parametrization of BCMs with increased collection of both behaviour and disease-related information. For instance, Van Kerckhove *et al*. [198] studied social contact patterns of symptomatic ILI cases during the pandemic. We encourage the collection of such real-time data in future outbreaks to guide policy-makers in the establishment of an optimal response strategy. For some models, data are just not available, and one needs to resort to assumptions to model behavioural change. Note also that excluding behavioural change from infectious disease models equates to assuming behaviour is unaffected by risk perceptions and disease incidence, and vice versa. Ignoring behavioural responses in the face of substantial changes in risk perceptions is probably worse than making assumptions within a theoretical model in the first place. This review has also met with important limitations in clarity of assumptions and methods in many publications, notwithstanding transparency is an essential part of publishing credible and replicable research.

### 4.2. Disease-dependent model specification

We observed that the specification of BCMs largely depends on the disease being investigated and the prevention measures considered. Clearly, the transmission characteristics (e.g. air and saliva borne versus STIs), the potential prevention measures (e.g. social distancing versus condom use) and the epidemic stage (e.g. emergence versus endemic equilibrium versus elimination) are interdependent, and determine both the utility and specification of a BCM. For instance, many influenza models use vaccination as a prevention measure with individuals evaluating their previous influenza vaccination decisions to determine the current season's strategy. It would seem unrealistic to require more data to parameterize both behavioural change and disease transmission models with the aim to develop more general models that suit any infectious disease, albeit that behavioural change in response to one disease's risk perceptions could change the risk perceptions of another. At the current stage of BCM development and parametrization, generalized BCMs accommodating multiple pathogens and different transmission routes seem unrealistic. However, it would be easier to combine multiple diseases with the same transmission and prevention properties. For instance, BCMs assessing the combined effects of vaccination scares on MMR and diphtheria, tetanus, pertussis (DTP) disease seem intuitively possible and relevant, though technically challenging and high on data demands.

Developing BCMs with multiple prevention measures is also challenging. Again, we take influenza as an example where we discovered a multitude of prevention measures in our selection (also see table 1): vaccination, social distancing, pre-exposure prophylaxis by antivirals, hygiene measures and others. Interdependencies between these prevention strategies may occur. For instance, a person vaccinated for seasonal influenza may put less effort into hygienic measures such as hand-washing. However, individuals taking hygiene measures may also be more inclined to engage in social distancing if these individuals are more risk-averse. Researchers need to take into account that focusing health policy on one prevention measure may induce ‘crowding out’ of other prevention measures because of such interdependencies. Hence, it is useful to assess the total effect of combined prevention efforts when evaluating policies to reduce the incidence of a disease. Models introducing behavioural change with interdependencies between different prevention measures are influenced by both intrinsic and extrinsic factors.

The popularity of emergence-driven research has many drivers: often new research funding and data collection opportunities arise as an emergence unfolds for the development and parametrization of new models to inform health policy.

### 4.3. Social networks and individual-based modelling

We observed a rise in the number of studies using (complex) social networks and IBMs to represent disease spread and individual behavioural changes. Social network models impose a structure in the population enabling the identification of model subjects at the individual-level. The implementation of these networks creates a coherent environment to model: social distancing as a prevention measure, the spread and clustering of disease- and prevention-related information and disease dynamics itself. In addition, neighbours can be identified to implement game-theoretic models with imitation dynamics, potentially resulting in clustering of prevention measures. It is clear that the development of these networks has increased the feasibility of modelling local or combined local–global information sources in a BCM. Nevertheless, the selection of an individual-level model is often a trade-off between the desirability for heterogeneity and IBM-specific hurdles such as the computational burden, greater risk of coding errors and potential loss of transparency and reproducibility. Here too, data availability is key to develop relevant models. For example, one could use the POLYMOD study on mixing patterns to construct a synthetic population or a network [199]. Still, more research is needed to enrich the validity of synthetic populations as a representation of real-life dynamics. We refer to a review by Wang *et al*. [200] focusing on coupling disease dynamics with behaviour in complex networks. A more general work covering BCMs is the book by Manfredi & D'Onofrio [201].

Some models use a single social network for both the disease transmission process and the formation of behaviour. Nonetheless, depending on the background, separate networks may be needed to model the spread of risks and the spread of information influencing behaviour. Take for instance anti-vaccine sentiments. These are often spread through blogs, Facebook groups and other social media [197]. Unlike these sentiments, infections are not spread through the Internet, and as a result require an additional network of physical contacts (see also Grim *et al*. [202], who make the case for modelling multiple networks). Additionally, the timescale of disease transmission can differ substantially from that of information spread leading to behaviour change. The models by Fukuda *et al*. [31], Helbing *et al*. [167] and Maharaj & Kleczkowski [134] are useful examples to guide further development of BCMs with separate parallel and sometimes interacting networks.

### 4.4. Internet and social media

Information gathering by individuals has evolved over the past decades with the introduction of the Internet, mobile phones and associated social media applications. It is well documented that web-based information can provide a distorted picture about disease risks and adverse events from vaccinations [203–205]. For instance, the search term ‘MMR vaccine’ in Google is automatically complemented by the suggestions ‘autism’ or ‘side effects’. We know individuals retrieve information using these sources for disease-related or prevention-related information and as a result, individuals are exposed to a wide variety of biased information. We recommend policy-makers to implement measures to help individuals to distinguish between evidence-based and unsubstantiated information. A quality label for health-related websites and public health information campaigns are two examples of such measures. Surveys can help understanding how individuals form their perceptions and where they obtain their information.

Another challenge we are faced with, given the popularity of social media, is whether we can still make a distinction between global and local information and how to use these sources of information to construct BCMs. We motivate by example: are Tweets local or global information? In essence, this information can be accessed by anyone, so that they are global. However, at the same time, Tweets are primarily shared among contacts that ‘follow’ each other, which defines local information. In addition, Facebook contacts are not necessarily close in a geographical sense, such that ‘local’ relates more to the possibility of clustering, moving beyond geography. This evolution reinforces the need for having distinct networks in the same model. While social media require reconsidering how information spread is modelled, they also present an opportunity to gather data on behaviour and behavioural changes. A number of studies we identified already integrated social media data [25,60,66,197]. We expect future modelling studies to increasingly use social media as a data source to parametrize BCMs.

### 4.5. Irrational behaviour and altruism

BCMs have evolved from the perspective of a fully rational ‘Homo economicus’ to a more reasonable, empathic ‘Homo sapiens’. This evolution is conform the findings of surveys examining individuals' drivers to take vaccination [9,192,193] and common sense in general. The study of Shim *et al*. [23] even considers altruism explicitly as a driver of individuals to take vaccination. In the most recent literature, only few papers are still using a pure, self-centred game-theoretic model. Instead, in the majority of the papers, some form of irrational behaviour has been introduced by the inclusion of social influences or imitation. It is striking, however, that most of the imitation BCMs did not empirically justify their choice of stickiness parameter.

### 4.6. Level of detail of behaviour

Many BCMs today capture, to some extent, heterogeneity in behaviour; individual-level networks can, for instance, introduce heterogeneity in the number of neighbours that can influence a person to adopt preventive measures. Some population models split the population into compartments representing different levels of risk attitude [89]. Some IBMs introduce personal experiences with disease or prevention measures in behaviour change models [33].

Moreover, heterogeneity in behaviour can be split into two categories: heterogeneity in information an individual receives (e.g. the social contact network of the individual) and heterogeneity in the response to this information (e.g. assigning individual values of stickiness of response in models with imitation). The majority of the publications include individual heterogeneity as the information they are exposed to, whereas only few include the latter category.

The desirability of heterogeneity in behaviour depends on the circumstances and characteristics observed. We illustrate by example: for measles in a highly vaccinated population, it has been observed that unvaccinated individuals and anti-vaccine sentiments are clustered and, as a result, heterogeneity in behaviour should be introduced in behaviour models. For example, one can introduce a distinction between vaccine sceptics and vaccine believers [90].

Again, the availability of real-life observations determines to a large extent the feasibility of introducing heterogeneity in BCMs. Why develop a complex model with large heterogeneity if the parameters cannot be informed by real-life observations? A trade-off needs to be made in terms of computational efficiency, data availability and desirability of heterogeneity given the context of the disease [15].

### 4.7. Limitations and strengths

Our search was limited to the past 6 years. However, a previous review ended where we start, and since this field is transitioning fast with rapidly increasing computational and research capacity, we believe the most recent years are the most informative. This is also testified by the evolution of our search yield over the 6 year period we covered. Our strength lies in the transparent and systematic way we have searched and analysed the literature according to the standards of systematic review. Nevertheless, as with any systematic review, our search string strikes a balance between completeness and feasibility. Given the current lack of a consistently used common term for the models we review, it is inevitable that we missed some admissible research. Indeed, it came to our attention that, for instance, [206–208] terms were not retrieved by our search, although they would satisfy our eligibility criteria. This emphasizes the need for a specific terminology. We therefore propose the use of the term ‘behavioural change model’ in title, abstract or keywords to facilitate more accurate identification of relevant studies by researchers in different fields.

## 5. Conclusion

We have systematically reviewed the literature on BCMs published from 2010 until 2015. We analysed and classified 178 references after full-text processing. We proposed a classification of the BCMs based on the decision-making process of the individual. We can summarize our findings in line with the six aims we listed in the introduction. Regarding the technological advancements and increased data availability (i), we find that social media and big data are useful to parametrize BCMs and present an as yet insufficiently explored source of information. Social media can, however, introduce a bias in individuals' prevention- or disease-related perceptions. In addition to the health recommendations they make, policy-makers can optimize their influence by enabling the collection and accessibility of government-owned data (such as surveillance) and by establishing a quality label for disease-related websites. Further, we can confirm that behavioural immunity is often contingent on the disease (ii): BCMs are disease and situation-dependent, which we strongly support. Regarding model validation and parametrization with quantifiable observations (iii), we can state that additional data sources are needed to specify relevant BCMs. Although the 2009 influenza pandemic presented an opportunity for parametrization and validation of both disease transmission and BCMs for flu-like illnesses, there is still much room for improvement in other disease areas. Current models have, without a doubt, assessed the importance of social networks in individual decisions (iv). Individual-level models such as IBMs are extremely useful to tackle behaviour changes and to mimic disease transmission better. More specifically, (v) the diversity observed in BCMs has increased the feasibility of introducing social influences and irrational behaviour (vi). In terms of policy recommendations, it is highly important to think about the total effect of an intervention, with possible implications on all prevention strategies.

The expansion of BCMs has been remarkably valuable. We encourage researchers to incorporate behaviour changes in future disease transmission models and to be transparent about the assumptions they make if data sources for parametrization or validation are sparse.

## Supplementary Material

References Database
